# The HLA-DRB1*09:01-DQB1*03:03 haplotype is associated with the risk for late-onset Alzheimer’s disease in *APOE* ${{\varepsilon }}$4–negative Japanese adults

**DOI:** 10.1038/s41514-023-00131-3

**Published:** 2024-01-02

**Authors:** Daichi Shigemizu, Koya Fukunaga, Akiko Yamakawa, Mutsumi Suganuma, Kosuke Fujita, Tetsuaki Kimura, Ken Watanabe, Taisei Mushiroda, Takashi Sakurai, Shumpei Niida, Kouichi Ozaki

**Affiliations:** 1 Medical Genome Center, Research Institute, National Center for Geriatrics and Gerontology, Obu, Aichi 474-8511 Japan; 2 RIKEN Center for Integrative Medical Sciences, Yokohama, Kanagawa 230-0045 Japan; 3 Department of Cardiovascular Medicine, Hiroshima University Graduate School of Biomedical and Health Sciences, Hiroshima, 734-8551 Japan; 4 Department of Prevention and Care Science, Research Institute, National Center for Geriatrics and Gerontology, Obu, Aichi 474-8511 Japan; 5 Japan Society for the Promotion of Science, Tokyo, 102-0083 Japan; 6 NCGG Biobank, National Center for Geriatrics and Gerontology, Obu, Aichi 474-8511 Japan; 7 Core Facility Administration, Research Institute, National Center for Geriatrics and Gerontology, Aichi, 474-8511 Japan

**Keywords:** Genome, Risk factors, Biomarkers, Alzheimer's disease

## Abstract

Late-onset Alzheimer’s disease (LOAD) is the most common cause of dementia among those older than 65 years. The onset of LOAD is influenced by neuroinflammation. The human leukocyte antigen (HLA) system is involved in regulating inflammatory responses. Numerous HLA alleles and their haplotypes have shown varying associations with LOAD in diverse populations, yet their impact on the Japanese population remains to be elucidated. Here, we conducted a comprehensive investigation into the associations between LOAD and HLA alleles within the Japanese population. Using whole-genome sequencing (WGS) data from 303 LOAD patients and 1717 cognitively normal (CN) controls, we identified four-digit HLA class I alleles (A, B, and C) and class II alleles (DRB1, DQB1, and DPB1). We found a significant association between the HLA-DRB1*09:01-DQB1*03:03 haplotype and LOAD risk in *APOE* ${\rm{\varepsilon }}$4–negative samples (odds ratio = 1.81, 95% confidence interval = 1.38–2.38, *P* = 2.03${\times 10}^{-5}$). These alleles not only showed distinctive frequencies specific to East Asians but demonstrated a high degree of linkage disequilibrium in *APOE* ${\rm{\varepsilon }}$4–negative samples (*r*^2^ = 0.88). Because HLA class II molecules interact with T-cell receptors (TCRs), we explored potential disparities in the diversities of TCR α chain (TRA) and β chain (TRB) repertoires between *APOE* ${\rm{\varepsilon }}$4–negative LOAD and CN samples. Lower diversity of TRA repertoires was associated with LOAD in *APOE* ${\rm{\varepsilon }}$4-negative samples, irrespective of the HLA DRB1*09:01-DQB1*03:03 haplotype. Our study enhances the understanding of the etiology of LOAD in the Japanese population and provides new insights into the underlying mechanisms of its pathogenesis.

## Introduction

Alzheimer’s disease (AD) is the most common cause of dementia among the elderly population, and it is the most frequent multifactorial neurodegenerative disease^1,2^. Based on the age of onset, AD is classified into two types: early-onset AD (EOAD, onset < 65 years) and late-onset AD (LOAD, onset ≥ 65 years). The majority of AD cases are sporadic LOAD^3^, which presents as a heterogeneous disorder influenced by complex interactions of both genetic and environmental risk factors. The genetic heritability is estimated to be substantial, ranging between 60% and 80%^4^.

Genetic associations provide valuable insights into understanding the complex etiology of this disease. Genome-wide association studies (GWAS) and WGS studies have identified susceptibility loci linked to LOAD^5,6^, as well as rare variants associated with LOAD^7,8^. LOAD-associated variants are enriched in genes like *APOE*, *ABCA7, BIN1*, *CR1*, *CD33*, *CLU*, *CD2AP*, *PICALM*, *SORL1*, *TREM2*, and more^9^. These LOAD risk genes are associated with functions ranging from immune response (*CLU, CR1, CD33, ABCA7*, and *TREM2*) to endocytosis (*BIN1, PICALM*, and *CD2AP*) and lipid processing (*APOE, ABCA7*, and *SORL1*)^10,11^. Consequently, a substantial proportion of LOAD risk genes are intertwined with the immune system. Also, our recent GWAS study has identified two distinct groups of LOAD patients, with one group characterized by immune-related genes^12^. Moreover, neuroinflammation has emerged as a significant contributor to the onset and progression of LOAD^10,13^. Recent clinical observations have highlighted the involvement of neutrophils, essential components of the acute inflammatory response, in LOAD pathogenesis and cognitive impairment^14,15^.

The HLA system plays a pivotal role as a regulator of inflammatory responses, contributing to the understanding of LOAD-associated neuroinflammation^16^. The HLA complex is located on the short arm of chromosome 6 (6p21), which spans approximately 4000 kilobases of DNA containing more than 200 genes^17^. This region is divided into three major classes: class I (HLA-A, HLA-B, HLA-C), class II (HLA-DP, HLA-DQ, and HLA-DR), and class III (with most genes having unknown function)^18^. Numerous association studies have reported significant associations between HLA loci within the HLA class I and class II regions and LOAD. For instance, HLA-A2 alleles have been associated with LOAD risk by influencing hippocampal volume alterations^19^. HLA-A1 alleles and HLA-A24 alleles have been associated with delayed LOAD development in *APOE* ${\rm{\varepsilon }}$4-positive adults in Italy^20^ and *APOE* ${\rm{\varepsilon }}$4-negative members of the Han Chinese population^21^, respectively. HLA-DRB1 alleles (specifically DRB1*03 and DRB1*09) and HLA-DQB1*06 alleles have been reported to be associated with LOAD risk in German^22^ and Iranian^23^ populations, respectively. In addition, many HLA genes are inherited as haplotypes with varying frequencies in different populations^24^, such as the HLA-DRB1*15:01-DQB1*06:02 and HLA-DRB1*04:02-DQB1*03:02 haplotypes in Tunisians^25^ and the HLA*-*A*03*:*01-B*07*:*02-DRB1*15*:*01-DQA1*01*:*02-DQB1*06*:*02 haplotype in Europeans^24^. Despite these findings, the associations between HLA alleles and LOAD risk in the Japanese population have yet to be established.

Here, we conducted a comprehensive investigation into the associations between LOAD and HLA alleles within the Japanese population. Using WGS data from a large number of older Japanese people, we identified four-digit HLA class I alleles (A, B, and C) and class II alleles (DRB1, DQB1, and DPB1). Our analysis revealed an association between the HLA-DRB1*09:01-DQB1*03:03 haplotype and LOAD risk in *APOE* ${\rm{\varepsilon }}$4-negative samples. These alleles not only showed distinctive frequencies specific to East Asians but demonstrated a high degree of linkage disequilibrium in *APOE* ${\rm{\varepsilon }}$4-negative samples. Because HLA class II molecules interact with TCRs, we further explored potential disparities in the diversities of TRA and TRB repertoires between samples from *APOE* ${\rm{\varepsilon }}$4-negative LOAD and CN adults. We revealed that lower diversity of TRA repertoires was associated with LOAD in *APOE* ${\rm{\varepsilon }}$4-negative samples, irrespective of the HLA DRB1*09:01-DQB1*03:03 haplotype.

## Results

### WGS subjects

A total of 2020 samples from Japanese donors, consisting of 303 LOAD patients and 1717 CN controls, were used in this study. All of the donors were aged at least 65 years. Ages were similar between groups, with a mean of 75 years for the LOAD group and 77 years for the CN group. The donors were further divided into whether they were positive or negative *APOE* ${\rm{\varepsilon }}$4 allele. Each category had more samples from females than from males, with a large difference in the LOAD group than the CN group (Table 1).

**Table 1:** Characteristics of patients whose samples were used for WGS.

Type	Phenotype	Number of samples	Age (mean ± 1 SD)	Female:male
All	LOAD	303	74.6 $\pm$6.3	1.53:1
	CN	1717	76.7 $\pm 3.0$	1.14:1
*APOE* $\varepsilon$4-positive	LOAD	108	75$.2\pm 7$.0	1.63:1
	CN	326	76.5 $\pm$3.7	1.01:1
*APOE* $\varepsilon$4-negative	LOAD	195	74.3 $\pm 5.8$	1.47:1
	CN	1391	76.8 $\pm 4.0$	1.18:1

*CN* cognitively normal controls, *LOAD* late-onset Alzheimer’s disease, *SD* standard deviation.

### Associations of HLA alleles

By using HISAT-genotype software^26^, we identified a total of 251 four-digit HLA alleles from the 2020 WGS samples. Among these alleles, 93 were classified as common alleles with allele frequency ≥ 0.01 (HLA-A = 13 alleles, HLA-B = 21 alleles, HLA-C = 13 alleles, HLA-DRB1 = 20 alleles, HLA-DQB1 = 12 alleles, and HLA-DPB1 = 14 alleles). Associations of these alleles with LOAD were assessed by using logistic regression, adjusting for sex and age. However, none of these alleles demonstrated a statistically significant difference in allele frequency between LOAD and CN subjects (Supplementary Figure 1).

**Fig. 1:**
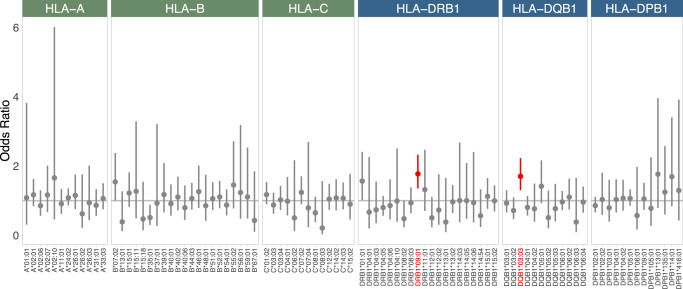
Associations of HLA class I and class II alleles with LOAD in *APOEε*4-negative subjects. HLA class I (A, B, and C) and class II (DRB1, DQB1, and DPB1) alleles were obtained. The associations of high-resolution four-digit HLA alleles were assessed by using logistic regression with adjustment for sex and age. A false discovery rate (FDR) was calculated by using the Benjamini–Hochberg method. Red bars represent HLA alleles at an FDR < 0.05. Error bars represent 95% confidence intervals.

We next examined the associations of the alleles separately for *APOE* ${\rm{\varepsilon }}$4-positive and *APOE* ${\rm{\varepsilon }}$4-negative subjects. While no statistically significant difference between LOAD and CN subjects were observed among *APOE* ε4-positive subjects (Supplementary Figure 2), a distinct pattern emerged for *APOE* ${\rm{\varepsilon }}$4-negative subjects. We discovered that the allele frequencies of HLA-DRB1*09:01 and HLA-DQB1*03:03 were significantly higher in LOAD patients than in CN subjects with an adjusted *P* value < 0.05 (Fig. 1 and Table 2). Neither the HLA-DRB1*09:01 nor the HLA-DQB1*03:03 allele showed significant deviations from Hardy–Weinberg equilibrium, as indicated by *P* values of 0.27 and 0.51, respectively.

**Table 2:** Frequencies of HLA-DRB1*09:01 and HLA-DQB1*03:03 alleles.

Subjects	HLA alleles (A1)	Number of samples (A1/A1A2/A2) A1 AF				OR	95% CI	P*	*P*_adj_
		LOAD	CN	LOAD	CN				
*APOE* $\varepsilon$4-negative	DRB1*09:01	13/58/124	27/331/1033	0.22	0.14	1.77	1.35–2.33	3.32$\times {10}^{-5}$	8.63$\times {10}^{-4}$
	DQB1*03:03	13/60/122	30/355/1006	0.22	0.15	1.70	1.30–2.23	0.0001	0.001
*APOE* $\varepsilon$4-positive	DRB1*09:01	1/25/82	7/88/231	0.13	0.16	0.82	0.51–1.31	0.40	0.57
	DQB1*03:03	1/24/83	9/93/224	0.12	0.17	0.71	0.45–1.15	0.16	0.25

*A2* non-A1 allele, *AF* allele frequency, *LOAD* Late-onset Alzheimer’s disease, *CN* cognitively normal controls, *OR* odds ratio of LOAD vs. CN, *CI* confidence interval, *P*_*adj*_ adjusted *P* value.

*P values were obtained from logistic regression with adjustment for age and sex.

### Validation of HLA alleles and allele frequencies of the alleles

To validate the accuracy of next-generation sequencing (NGS)-based HLA alleles, genotyping for the HLA-DRB1:09:01 allele was conducted through experimental validation at the HLA Laboratory, NPO (Kyoto, Japan). Twenty-five samples, comprising 10 homozygotes and 10 heterozygotes possessing the HLA allele of interest, along with five homozygotes lacking the HLA allele, were randomly selected from *APOE* ${\rm{\varepsilon }}$4-negative samples. Of the 50 alleles assessed, 49 were consistent with our NGS-based HLA alleles, resulting in a high concordance rate of 0.98 (Supplementary Table 1).

**Fig. 2:**
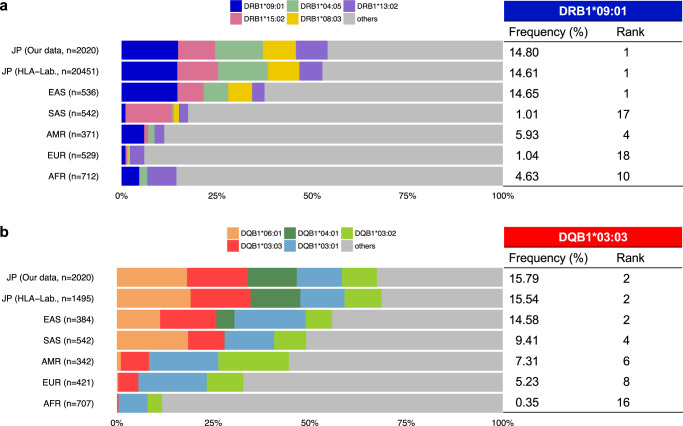
Allele frequencies of HLA-DRB1*09:01 and HLA-DQB1*03:03 in several populations. Allele frequencies of HLA-DRB1*09:01 (**a**) and HLA-DQB1*03:03 (**b**) in six populations were obtained from three datasets: our WGS data, the HLA Laboratory, and the 1000 Genome Project. The five top-ranked HLA-DRB1 (**a**) and HLA-DQB1 (**b**) alleles in our WGS data are represented, and the HLA-DRB1*09:01 (**a**) and HLA-DQB1*03:03 (**b**) are shown in blue and red, respectively. JP Japanese; EAS East Asian, SAS South Asian, AMR admixed American, EUR European, AFR African, n number of samples.

The allele frequencies of HLA-DRB1*09:01 and HLA-DQB1*03:03 were found to be 14.80% and 15.79%, respectively, within our Japanese WGS dataset. These frequencies closely matched those obtained from a substantial collection of healthy Japanese samples provided by the HLA Laboratory, NPO (Kyoto, Japan) (HLA-DRB1*09:01 = 14.61%, HLA-DQB1*03:03 = 15.54%, Fig. 2). We also compared our results with allele frequencies from various populations in data provided by the 1000 Genomes Project. Both HLA alleles, DRB1*09:01 and DQB1*03:03 had a high occurrence rate, particularly within the East Asian population, including Japan. DRB1*09:01 ranked first in this population, and DQB1*03:03 ranked second (Fig. 2).

### HLA-DRB1*09:01-DQB1*03:03 haplotype

Since the allele frequencies of HLA-DRB1*09:01 in LOAD and CN subjects showed similar frequencies of those of HLA-DQB1*03:03 in LOAD and CN subjects (Table 2), we examined the linkage disequilibrium (LD) of these alleles. Our finding revealed a high LD within *APOE* ε4-negative subjects, with *r*^2^ values of 0.88. Subsequently, we examined whether the haplotype formed by these alleles was associated with the risk for LOAD. Our analysis yielded significant results, with the haplotype’s allele frequency being considerably higher in LOAD patients than in CN subjects (odds ratio = 1.81, 95% confidence interval = 1.38 to 2.38, Table 3). Furthermore, we examined the associations between the number of haplotypes and Mini-Mental State Examination (MMSE) scores, separately for *APOE* ${\rm{\varepsilon }}$4-positive and *APOE* ${\rm{\varepsilon }}$4-negative LOAD patients. The associations were evaluated by using linear regression analysis, with adjustments for sex and age. However, we found no statistically significant differences between them (*APOE* ${\rm{\varepsilon }}$4-positive LOADs, *P* = 0.96; *APOE* ${\rm{\varepsilon }}$4-negative LOADs, *P* = 0.12).

**Table 3:** Frequencies of the HLA-DRB1*09:01-DQB1*03:03 haplotype.

Subjects	Number of samples (A1/A1A2/A2) A1 AF				OR	95% CI	P*
	LOAD	CN	LOAD	CN			
*APOE* $\varepsilon$4-negative	13/57/125	25/326/1040	0.21	0.14	1.81	1.38–2.38	2.03$\times {10}^{-5}$
*APOE* $\varepsilon$4-positive	1/24/83	7/87/232	0.12	0.16	0.79	0.49–1.28	0.34

*A1* HLA-DRB1*09:01-DQB1*03:03 haplotype, *A2* non-A1 haplotype, *AF* allele frequency, *LOAD* Late-onset Alzheimer’s disease, *CN* cognitively normal controls, *OR* odds ratio of LOAD vs. CN, *CI* confidence interval.

*P value was obtained from logistic regression with adjustment for age and sex.

Kawabata et al. previously reported that people homozygous for the DRB1*09:01-DQB1*03:03 haplotype were more frequent among Japanese with type 1 diabetes than in control subjects^27^. We therefore checked whether this specific haplotype was also associated with diabetes in *APOE* ${\rm{\varepsilon }}$4-negative Japanese people. We selected a subset of 1586 samples from *APOE* ${\rm{\varepsilon }}$4-negative subjects, including 53 patients with diabetes and 166 without diabetes (controls) according to defined diabetes criteria (see “Methods”). Within the diabetes patient group, there were three haplotype homozygotes, 21 haplotype heterozygotes, and 29 non-haplotype homozygotes, although the diabetes patients aged $\ge$65 years were more likely to have type 2 diabetes. The control group consisted of 10 haplotype homozygotes, 40 haplotype heterozygotes, and 116 non-haplotype homozygotes. We conducted an association analysis between the haplotype and diabetes/control samples by using Fisher’s exact test. However, there was no statistically significant association in recessive model (*P* > 0.99, Supplementary Table 2). We concluded that the HLA-DRB1*09:01-DQB1*03:03 haplotype was not associated with diabetes within our *APOE* ${\rm{\varepsilon }}$4-negative Japanese population.

### Clonal diversity of TCR repertoires

HLA class II molecules, including the HLA-DRB1 and HLA-DQB1 variants present in our haplotype, play a crucial role in interacting with TCRs. Given this interaction, we explored the potential impact of our identified haplotype on the diversities of TCR repertoires within *APOE* ${\rm{\varepsilon }}$4-negative Japanese adults. TRUST4 software^28^ was used to estimate the diversities of TRA and TRB repertoires from bulk RNA-sequencing (RNA-seq) within each sample (see “Methods”). Our dataset comprised 114 RNA-seq samples derived from *APOE* ${\rm{\varepsilon }}$4-negative subjects (55 LOAD and 59 CN samples) obtained from the National Center for Geriatrics and Gerontology (NCGG) Biobank database. A logistic regression model was used to identify statistically significant increases or decreases in the clonal diversity of TCR repertoires between phenotypes with adjustments for age and sex and for age, sex, and our specific haplotype (i.e., DRB1*09:01-DQB1*03:03). Despite the lack of statistically significant differences in the TRB repertoires under either adjustment, the diversity of the TRA repertoires did demonstrate statistically significant decreases under both adjustments in *APOE* ${\rm{\varepsilon }}$4-negative samples (Table 4). These findings provided insights into the associations between the diversities of TRA repertoires and the risk for LOAD within the *APOE* ε4*-*negative Japanese population, regardless of the presence of the HLA DRB1*09:01-DQB1*03:03 haplotype.

**Table 4:** Clonal diversity of TCR repertoires between phenotypes in *APOE* ${\varepsilon }$4-negative subjects.

TCR type	Adjustment	OR	95% CI	P*
TRA	Age + Sex	0.983	0.966–0.998	0.034
	Age + Sex + haplotype^a^	0.982	0.966–0.998	0.033
TRB	Age + Sex	0.984	0.965–1.002	0.097
	Age + Sex + haplotype^a^	0.995	0.966–1.003	0.11

*TCR* T-cell receptor, *TRA* T-cell receptor alpha, *TRB* T-cell receptor beta, *OR* odds ratio of LOAD vs. CN, *CI* confidence interval.

*P values were obtained from logistic regression.

^a^Haplotype: HLA-DRB1*09:01-DQB1*03:03.

## Discussion

Through our investigation involving WGS data from 2020 Japanese people, a specific HLA class II haplotype, HLA-DRB1*09:01-DQB1*03:03, emerged as being associated with the underlying mechanisms of LOAD. Although both HLA-DRB1 and HLA-DQB1 alleles are commonly observed in East Asian populations, their allele frequencies vary across different ethnic populations. Within the HLA-DQB1 locus, the HLA-DQB1*03:01 allele is frequently observed across all populations, whereas the HLA-DQB1*06:01 allele is particularly common among Asian populations. Our identified HLA-DQB1*03:03 allele had the second highest allele frequency after the HLA-DQB1*06:01 allele among Japanese adults. In the HLA-DRB1 locus, the HLA-DRB1*09:01 allele was the most frequent within East Asian populations, accounting for approximately 15%. This stands in contrast to South Asian, American, and European populations, where the HLA-DRB1*07:01 allele is more prominent (SAS = 19%, AMR = 10%, EUR = 14%). In Africans, HLA-DRB1*15:03 was the most frequent, accounting for approximately 12%. The HLA-DRB1*15:03 allele, dominant in Africans, is not present in East Asian, South Asian, or European populations. Many LOAD-associated HLA alleles have been reported in similar studies^20,21,24,25^, but our findings were not included in their results due to their East Asian specificity. This result indicates the significance of population-specific variations in HLA allele frequencies and highlights the importance of region-specific investigations in understanding complex diseases like LOAD.

Various methods for HLA genotyping using NGS data have been developed^26,29,30^ and evaluated for their concordance rates, particularly at 2-digit and 4-digit allele resolutions^31^. In this study, we employed a highly accurate NGS-based HLA genotyping method called HISAT-genotype. Although the method has demonstrated an extremely high concordance rate (≥ 0.97) at the 2-digit allele level (e.g., HLA-A*01), the concordance rate at the 4-digit allele resolution has been slightly lower, especially for the HLA-DRB1 gene (with a concordance rate of 0.87)^26^. Because our findings included an HLA-DRB1 locus, we experimentally validated our HLA-DRB1 alleles. The concordance rate of these alleles was significantly higher than we expected, reaching 0.98. This outcome provides robust support for the reliability and validity of NGS-based HLA genotyping, suggesting its suitability for potential application in clinical use.

Our study has revealed that the DRB1*09:01-DQB1*03:03 haplotype is associated with the risk of LOAD in *APOE* ε4-negative Japanese adults. This haplotype has previously been linked to certain diseases in the Japanese population, such as type 1 diabetes^27^ and myeloperoxidase-antineutrophil cytoplasmic antibody-positive vasculitis (MPO-AAV)^32^. Although *APOE* ${\rm{\varepsilon }}$4-negative Japanese diabetes patients aged $\ge$ 65 years were more likely to have type 2 diabetes, our findings did not show a statistically significant association between the haplotype and the diabetes in *APOE* ${\rm{\varepsilon }}$4-negative Japanese people. However, further HLA genotyping and association studies in a larger number of samples may reveal a relationship between the HLA haplotype and diabetes risk. MPO-AAV is a life-threatening autoimmune disease which causes severe inflammation and destruction of small blood vessels, mainly in the kidneys of people older 50 years^33^. Given that neuroinflammation significantly contributes to the onset and progression of LOAD^10,13^, this result suggests that the presence of the DRB1*09:01-DQB1*03:03 haplotype might confer an increased risk of LOAD, even in adults without the *APOE* $\varepsilon$4 allele.

Indeed, our investigation into the interaction between the DRB1*09:01-DQB1*03:03 haplotype and the diversity of TCR repertoires indicated that a low diversity of TRA repertoires is associated with LOAD onset in *APOE* ε4-negative Japanese adults, providing valuable insights into the potential mechanisms underlying the association between this haplotype and LOAD risk in *APOE* ε4-negative Japanese people. Xu et al. previously reported that the diversity of TCR repertoires was significantly lower in samples from AD patients than in samples from CN individuals, as analyzed with single-cell RNA-seq^34^. Our previous study also reported that the diversity of B-cell receptor repertoires (IGH and IGK) and TRA were significantly smaller in people with LOAD progression, using a large number of RNA-seq datasets (> 850 Japanese samples)^35^. These results suggest that the DRB1*09:01-DQB1*03:03 haplotype and the diversity of TCR repertoires could potentially be associated with the onset of LOAD through different mechanisms. However, given that HLA Class II molecules interact with CD4+ helper T-cells, we may need to investigate the association between this specific haplotype and the diversity of specific CD4+ helper TCR repertoires in *APOE* ε4-negative Japanese adults in the near future.

To our knowledge, this study represents the largest NGS-based HLA genotyping analysis in Japanese LOAD cases. However, further investigations using larger sample sizes are likely to reveal additional pathogenic HLA alleles associated with LOAD. Our findings contribute to enhancing the understanding of LOAD and provide insight into its pathogenic mechanisms for future investigations. While our current dataset may not yet be sufficient to comprehensively identify all HLA alleles associated with LOAD in the Japanese population, undertaking an association study between LOAD and CN through a large number of WGS samples will undoubtedly lead to the discovery of new HLA alleles associated with LOAD.

## Methods

### Ethics statements

This study received approval from the ethics committee of the National Center for Geriatrics and Gerontology. The design and execution of the current study, which involved human subjects, were clearly described in a research protocol. Participation in the NCGG Biobank was voluntary, and all participants provided written informed consent before enrolling. Surrogate consent was obtained for patients with advanced cognitive decline.

### Subjects

All of the blood samples used in this study and the associated clinical data were obtained from the NCGG Biobank. Of the 2020 samples, 303 were from LOAD patients and the remaining 1717 were from CN subjects. Donors with LOAD were diagnosed with AD according to the criteria established by the National Institute on Aging—Alzheimer’s Association workgroups^36,37^. CN subjects had subjective cognitive complaints but normal cognition on a neuropsychological assessment with a comprehensive neuropsychological test and a MMSE score > 23. Diagnosis of all AD in all donors whose samples were used was based on medical history, physical examination and diagnostic tests, neurological examination, neuropsychological tests, and brain imaging with magnetic resonance imaging or computerized tomography. Diagnoses were made by experts, including neurologists, psychiatrists, geriatricians, and neurosurgeons, all of whom were experts in dementia and familiar with its diagnostic criteria. All of the samples were from adults 65 years of age or older.

### WGS data analysis

DNA concentration was measured by using a PicoGreen DNA assay, and fragmentation of DNA was assessed with agarose gel electrophoresis. High-quality DNA was used for DNA libraries. A WGS library was constructed by using the TruSeq DNA PCR-Free Library Preparation Kit (Illumina, Inc., San Diego, CA, USA) in accordance with the manufacturer’s instructions. WGS was conducted at Macrogen Japan Corporation, Takara Bio Inc., and GENEWIZ Inc. The DNA was sequenced by using either the Illumina HiSeq X Ten or NovaSeq 6000 platform, generating paired-end reads of 151 base pairs in accordance with the manufacturer’s instructions. All data used in this study were obtained from the NCGG Biobank database^8^, where they were initially registered.

### HLA genotyping from WGS data

NGS-based HLA genotyping was performed by using HISAT-genotype software^26^, which is accessible through a public GitHub repository (1.3.2 release; https://daehwankimlab.github.io/hisat-genotype/). Individual HLA genotyping was conducted by using two programs: “hisatgenotype” and “hisatgenotype_toolkit”. These programs facilitated the determination of HLA class I alleles (A, B, and C) and class II alleles (DRB1, DQB1, and DPB1). For case-control association studies, the high-resolution four-digit HLA alleles were employed. The associations were evaluated by using logistic regression analysis, with adjustments for sex and age. Adjusted *P* values were calculated with the Benjamini–Hochberg method. An adjusted *P* value of 0.05 or less was considered statistically significant. Logistic regression analysis (–logistic) and Hardy–Weinberg equilibrium test (–hardy) for HLA loci were performed by using PLINK software (version 1.90b)^38^. Allele frequencies of HLA loci in various populations were available publicly at https://hla.or.jp/med/frequency_search/ja/allele/ (the HLA Laboratory, NPO) and at http://ftp.1000genomes.ebi.ac.uk/vol1/ftp/data_collections/HLA_types/ (the 1000 Genomes Project).

### HLA haplotype

Beagle software (version 3.0.4)^39^ was used to phase HLA alleles into individual HLA haplotypes. This process was facilitated by employing two programs, “linkage2beagle.jar” and “beagle.jar”. The software package can be downloaded from http://faculty.washington.edu/browning/beagle/recent.versions/beagle_3.0.4_05May09.zip. To assess the degree of linkage disequilibrium between two HLA loci, *r*^2^ values were calculated by using PLINK software and the (–ld) parameter.

### Incident diabetes

Incident diabetes was identified through the following criteria: self-report of diabetes, usage of antihyperglycemic medicines, or hemoglobin A1c (HbA1c) levels ≥ 6.5%^40^. Conversely, subjects whose records lacked self-report of diabetes and antihyperglycemic medicine use while displaying HbA1c < 6.5%, were classified as not having diabetes (i.e., control samples). The examination of haplotype associations between diabetes and control samples were carried out with Fisher’s exact test, facilitated by the PLINK software with the (–model fisher) parameter^38^.

### Detection of T-cell receptor repertoires

All RNA-seq data were downloaded from the NCGG Biobank database^15^. TCR repertoires were identified from RNA-seq data using TRUST4 software (v1.0.5). This included the inference of CDR3 clonotypes, encompassing both the TRA and TRB. To estimate the clonal diversity of TCRs, an inverse Simpson index was employed, and the calculation was conducted by using VDJtools^41^ (v1.2.1). Assessments regarding the associations of the clonal diversity within TCR repertoires were conducted through logistic regression analysis between phenotypes, which were implemented with the R programming language (R Development Core Team, http://www.r-project.org/).

### Reporting summary

Further information on research design is available in the Nature Research Reporting Summary linked to this article.

### Supplementary information

Supplementary Information

reporting summary

## Data Availability

All datasets used or analyzed are available from the corresponding author on reasonable request.

## Code availability

We used the open-source program languages R (version 3.4.1) and Ruby (version 2.4.0) to analyze data and create plots. Code is available upon request from the corresponding authors.
