# Supplementary material for: The HLA-DRB1*09:01-DQB1*03:03 haplotype is associated with the risk for late-onset Alzheimer’s disease in APOE ε4–negative Japanese adults
Source: NPJ Aging. 2024 Jan 2;10(1):3. doi: 10.1038/s41514-023-00131-3 (PMC10761915; doi:10.1038/s41514-023-00131-3)
Supplement: Supplementary file 1 — Supplementary Information [file 41514_2023_131_MOESM1_ESM.pdf]

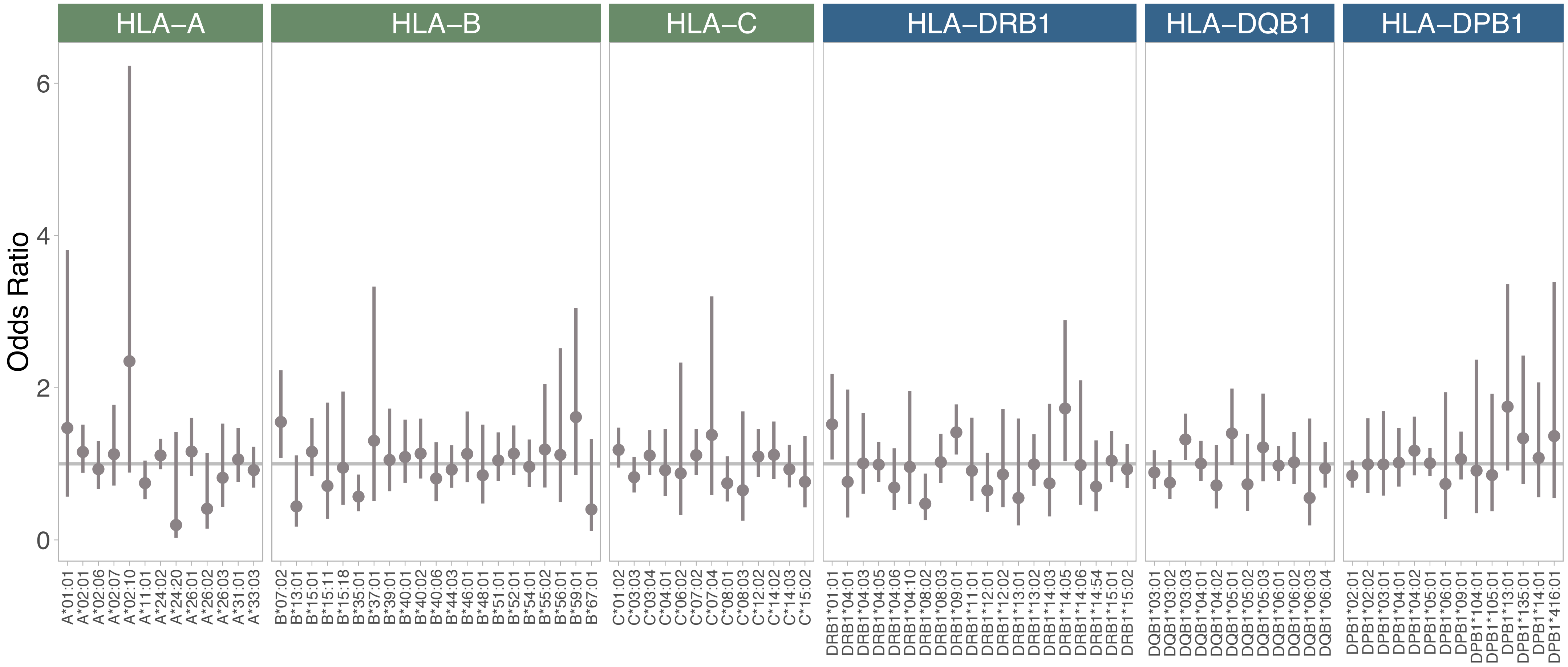

Supplementary Figure 1. Associations of HLA class I and class II alleles in all samples

HLA class I (A, B, and C) and class II (DRB1, DQB1, and DPB1) alleles were obtained. The associations of high resolution four-digit HLA alleles were assessed by using logistic regression with adjustment for sex and age. An FDR was calculated by using the Benjamini–Hochberg method. Red bars represent HLA alleles at an FDR < 0.05, but there were no significant differences in allele frequency between the LOAD and CN groups. Error bars represent 95% confidence intervals.

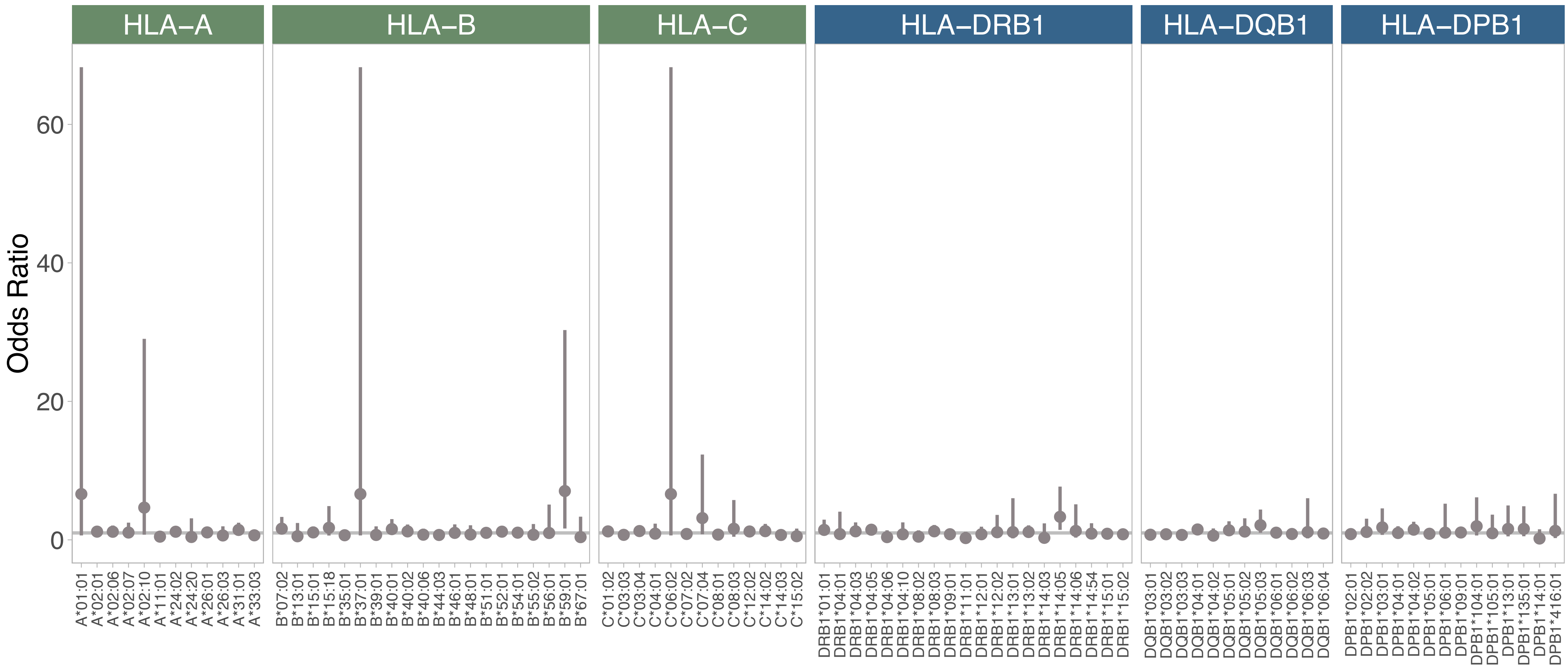

Supplementary Figure 2. Associations of HLA class I and class II alleles with LOAD in *APOE ε*4–positive subjects
HLA class I (A, B, and C) and class II (DRB1, DQB1, and DPB1) alleles were obtained. The associations of high resolution four-digit HLA alleles were assessed by using logistic regression with adjustment for sex and age. An FDR was calculated by using the Benjamini–Hochberg method. Red bars represent HLA alleles at an FDR < 0.05, but there were no significant differences in allele frequency between the LOAD and CN groups. Error bars represent 95% confidence intervals.

Supplementary Table 1. Validation of HLA*DRB1:09:01 alleles

Allele type	HISAT-genotype	HLA Laboratory
HLA*DRB1:09:01 homozygotes	DRB1*09:01 DRB1*09:01	DRB1*09:01 DRB1*09:01
	DRB1*09:01 DRB1*09:01	DRB1*09:01 DRB1*09:01
	DRB1*09:01 DRB1*09:01	DRB1*09:01 DRB1*09:01
	DRB1*09:01 DRB1*09:01	DRB1*09:01 DRB1*09:01
	DRB1*09:01 DRB1*09:01	DRB1*09:01 DRB1*09:01
	DRB1*09:01 DRB1*09:01	DRB1*09:01 DRB1*09:01
	DRB1*09:01 DRB1*09:01	DRB1*09:01 DRB1*09:01
	DRB1*09:01 DRB1*09:01	DRB1*09:01 DRB1*09:01
	DRB1*09:01 DRB1*09:01	DRB1*09:01 DRB1*09:01
	DRB1*09:01 DRB1*09:01	DRB1*09:01 DRB1*09:01
HLA*DRB1:09:01 heterozygotes	DRB1*04:07 DRB1*09:01	DRB1*04:07 DRB1*09:01
	DRB1*14:54 DRB1*09:01	DRB1*14:54 DRB1*09:01
	DRB1*08:03 DRB1*09:01	DRB1*08:03 DRB1*09:01
	DRB1*09:01 DRB1*08:03	DRB1*09:01 DRB1*08:03
	DRB1*04:01 DRB1*09:01	DRB1*04:01 DRB1*09:01
	DRB1*04:05 DRB1*09:01	DRB1*04:05 DRB1*09:01
	DRB1*04:03 DRB1*09:01	DRB1*04:03 DRB1*09:01
	DRB1*01:01 DRB1*09:01	DRB1*01:01 DRB1*09:01
	DRB1*11:01 DRB1*09:01	DRB1*11:01 DRB1*09:01
	DRB1*13:02 DRB1*09:01	DRB1*13:02 DRB1*09:01
non-HLA*DRB1:09:01 homozygotes	DRB1*13:02 **DRB1*12:57**	DRB1*13:02 **DRB1*13:02**
	DRB1*11:01 DRB1*01:01	DRB1*11:01 DRB1*01:01
	DRB1*15:02 DRB1*14:54	DRB1*15:02 DRB1*14:54
	DRB1*11:01 DRB1*01:01	DRB1*11:01 DRB1*01:01
	DRB1*04:05 DRB1*16:02	DRB1*04:05 DRB1*16:02

Mismatched alleles are shown in bold.

Supplementary Table 2. Association study of DRB1*09:01-DQB1*03:03 between diabetes and control samples

		No. of subjects		A1 AF		
Haplotype (A1)	Model	diabetes	control	diabetes	control	*P**
DRB1*09:01-DQB1*03:03	Recessive (A1/A1A2+A2)	3/50	10/156	0.06	0.06	1.00

Abbreviation: AF, allele frequency.

**P* values were obtained with Fisher's exact test.
